# Genomics pipelines to investigate susceptibility in whole genome and exome sequenced data for variant discovery, annotation, prediction and genotyping

**DOI:** 10.7717/peerj.11724

**Published:** 2021-07-26

**Authors:** Zeeshan Ahmed, Eduard Gibert Renart, Saman Zeeshan

**Affiliations:** 1 Institute for Health, Health Care Policy and Aging Research, Rutgers, The State University of New Jersey, New Brunswick, NJ, USA; 2 Department of Medicine, Robert Wood Johnson Medical School, Rutgers, The State University of New Jersey, New Brunswick, NJ, USA; 3 Cancer Institute of New Jersey, Rutgers, The State University of New Jersey, New Brunswick, NJ, USA

**Keywords:** Annotation, Bioinformatics, Genomics, Genotyping, Pipelines, Prediction, Variants, WGS, WES, Tools

## Abstract

Over the last few decades, genomics is leading toward audacious future, and has been changing our views about conducting biomedical research, studying diseases, and understanding diversity in our society across the human species. The whole genome and exome sequencing (WGS/WES) are two of the most popular next-generation sequencing (NGS) methodologies that are currently being used to detect genetic variations of clinical significance. Investigating WGS/WES data for the variant discovery and genotyping is based on the nexus of different data analytic applications. Although several bioinformatics applications have been developed, and many of those are freely available and published. Timely finding and interpreting genetic variants are still challenging tasks among diagnostic laboratories and clinicians. In this study, we are interested in understanding, evaluating, and reporting the current state of solutions available to process the NGS data of variable lengths and types for the identification of variants, alleles, and haplotypes. Residing within the scope, we consulted high quality peer reviewed literature published in last 10 years. We were focused on the standalone and networked bioinformatics applications proposed to efficiently process WGS and WES data, and support downstream analysis for gene-variant discovery, annotation, prediction, and interpretation. We have discussed our findings in this manuscript, which include but not are limited to the set of operations, workflow, data handling, involved tools, technologies and algorithms and limitations of the assessed applications.

## Introduction

Genetics is a field of biology, and it is about studying inherited variation, transmission, machinery, and processes of cellular reproduction. Genetic differences among Deoxyribonucleic acid (DNA) of the healthy and diseased individuals and populations are known as the variants. DNA structure was first solved in 1953 by Watson & Crick (1953), using crystallographic data generated by Rosalind Franklin and Maurice Wilkins (Zallen, 2003). It is a complex molecule, encapsulated within their cells including all information necessary to build and maintain an organism. Human DNA sequence is composed of coding and non-coding regions. Coding regions are the sequences that translate into protein, when non-coding are the regulatory sequences that do not encode into amino acids but can impact the degree, timing, and tissue specificity of gene expression. Different high-throughput technologies have been developed in the last few decades to sequence the DNA of human and several other species (Heather & Chain, 2016). So far, there have been three generations considered reporting progress in DNA sequencing.

First generation started with designing conceptual framework for DNA replication and encoding proteins in nucleic acids (Watson & Crick, 1953; Zallen, 2003), and then focused on sequencing RNA species (*e.g*., microbial ribosomal or transfer RNA, genomes of single-stranded RNA bacteriophages) (Heather & Chain, 2016). Different techniques were proposed, including but not limited to the measurement of nucleotide composition (Holley et al., 1961); detection of radiolabelled partial-digestion fragments (Sanger, Brownlee & Barrell, 1965); production of the first complete protein-coding gene sequence with 2-D fractionation (Min Jou et al., 1972; Fiers et al., 1976); DNA polymerase to synthesize from a primer using ‘plus and minus’ (Sanger & Coulson, 1975; Maxam & Gilbert, 1977); ‘chain-termination’ for DNA sequencing (Sanger, Nicklen & Coulson, 1977); and automated DNA sequencing with fluorometric based detection (Smith et al., 1985; Ansorge et al., 1986; Luckey et al., 1990; Hunkapiller et al., 1991). During second generation, luminescent method was used to measure pyrophosphate synthesis (Nyrén & Lundin, 1985); adapter sequences were introduced to bind libraries of DNA molecules with beads (Tawfik & Griffiths, 1998); Genome Analyzer (GA) machines were introduced for very short reads sequencing. Most importantly paradigm started shifting with the increased production of better-quality data using sequencing machines including, 454 (later bought by Roche and called as the GS 20), Solexa by Illumina, which then followed by the MiSeq, NextSeq, HiSeq (Voelkerding, Dames & Durtschi, 2009). Additionally, many other novel methodologies were also brought into the field *e.g*., sequencing by oligonucleotide ligation and detection (SOLiD) system from Applied Biosystems (McKernan et al., 2009), sequence-by-ligation by Complete Genomic (Drmanac et al., 2010), and post-light sequencing by Ion Torrent (Rothberg et al., 2011). Third generation has proven to be the most advanced (Schadt, Turner & Kasarskis, 2010; Niedringhaus et al., 2011; Gut, 2013), and equipped with high volume and much improved quality producing sequencing technologies, including single molecule sequencing (SMS) by Stephen Quake’s lab (Mardis, 2008), single molecule real time (SMRT) by Pacific Biosciences (Bragg et al., 2013), and solid-state technology to generate suitable nanopores by Oxford Nanopore Technologies (ONT) (Huse et al., 2007). Major differences among these generations and technologies are based on the depth of sequencing, number of produced DNA nucleotides, structure and overall size of the outcome (Mardis, 2008).

Clinical molecular laboratories are progressively discovering novel sequence variants for significantly regulated (up or down), expressed (high or low) and phenotype associated single and multiple genes linked to genetic disorders (Richards et al., 2015). The most popular DNA sequencing methodologies today are the whole-genome sequencing (WGS) and whole-exome sequencing (WES). The WGS is widely applied to sequence the entirety of the genome, while the WES is mainly used to only sequence the protein coding structures. Along with the production of in-depth and high-quality DNA sequence data (Zeeshan et al., 2020; Ahmed et al., 2019), one challenge that survived throughout three generations is the efficient processing of raw sequence data to support downstream analysis and clinical interpretation (Koboldt et al., 2010). It is still difficult to identify novel variants with high penetrance (Cavalleri & Delanty, 2012). Together with academic, commercial laboratories have also developed expertise to support genetic testing and implementing models of sequence variant interpretation (Pepin et al., 2016). Various bioinformatics tools have been developed worldwide to perform standalone and networking operations, which include cleansing of raw sequence data, converting raw signals into base calling, identifying regions of interest in genome, alignment, and assembly of contigs and scaffolds, and variant detection. Furthermore, well curated gene, variant (functional and non-functional), and disease annotation databases are available to support secondary and downstream sequence data analysis and interpretation (Ahmed et al., 2020b; Mailman et al., 2007; Tang et al., 2015). The scope of this study is to review and support the process of genetic testing with the classification of susceptibility genes to detect changes of clinical significance. Finding and interpreting such variations is still a challenge among diagnostic laboratories and clinicians (Ahmed & Ucar, 2017). We are interested in understanding and analyzing current state of genomics solutions for the identification of variants, alleles, and haplotypes by processing raw sequence data of variable length (Pabinger et al., 2014).

## Survey methodology

In this study, we consulted published literature and tested available material of the freely available bioinformatics pipelines. In this study we have assessed the set of operations, workflow, data handling, involved tools, technologies and algorithms, and limitations of different bioinformatic pipelines published in last 10 years and available through PubMed Central (PMC)—NCBI. We were focused on the standalone and networking bioinformatics applications proposed to efficiently process WGS and WES data, and support downstream analysis for gene-variant discovery, annotation, prediction, and interpretation. Based on our evaluation and conclusions drawn from comparative analysis, we have felt serious need of a solution addressing the current limitations to the field. As most of the Next Generation Sequencing (NGS) data processing and analytic application are based on the nexus of different command-line applications known as pipelines. Their execution not only requires good programming skills but deeper understanding of the computer science fundamentals *e.g*., UNIX commands, scripting, file management etc.

### Pipelines for WGS and WES data processing and variant calling

WGS and WES pipelines can be divided into three types: (I) cloud computing pipelines, (II) centralized pipelines, and (III) standalone pipelines. Cloud computing pipelines are mainly deployed in environments with on-demand compute resources managed and provided by externa vendors *e.g*., Amazon AWS, Google cloud, or Microsoft Azure. Centralized pipelines are developed to be deployed and executed in local computer. However, standalone pipelines are mostly applied in the high-performance computing environments (HPC). In this study, we have selected and evaluated eleven different WGS and WES pipelines of all three different types, which includes DNAp (Causey et al., 2018), STORMseq (Karczewski et al., 2014), ExScaliburn (Bao et al., 2015), Atlas2 (Evani et al., 2012), MC-GenomeKey (Elshazly et al., 2016), Simplex (Fischer et al., 2012), Whole Exome sequencing Pipeline web tool (WEP) (D’Antonio et al., 2013), SeqBench (Dander et al., 2014), VDAP-GUI (Menon et al., 2016), and fastq2vcf (Gao, Xu & Starmer, 2015).

**STORMseq** is a cloud-based pipeline for processing WES and WGS data (Karczewski et al., 2014). It is built as an Amazon Machine Image (AMI), which has all the information required to automatically launch an instance in the Amazon Web Services (AWS). It uses a graphical point-and-click interface for setting up the pipeline parameters. Once complete, all that is left is to upload the data to Amazon Compute cloud and deploy the pipeline. Authors have claimed it to be a pipeline that is customizable, user-friendly, and based on click-to-deploy architecture. Authors have developed STORMseq for mainly processing and analyzing short reads/sequences produced by the Illumina technology. Its operations include read cleansing and mapping to reference genome, removing duplicates, variant calling, and annotation. In read mapping step the FASTQ data is mapped to the reference genome, by using the Burrows-Wheeler Aligner (BWA) (Li & Durbin, 2009; Li & Durbin, 2010). and SNAP tools (Johnson et al., 2008). It is then followed by the read cleansing step, in which data is sorted and duplicates are removed to minimize the number of false positives. Genome Analysis Toolkit (GATK) (McKenna et al., 2010; Franke & Crowgey, 2020; Heldenbrand et al., 2019; Brouard et al., 2019; Poplin et al., 2017; Auwera et al., 2013; DePristo et al., 2011) is used for the variant calling step, in which Single Nucleotide Polymorphisms (SNPs) and insertions and deletions (INDELs) are called. In the final step variants are annotated using different reference databases and VEP tool (McLaren et al., 2016). Authors have validated STORMSeq (Karczewski et al., 2014) at two paired-end 100 bp read datasets (genome and exome) with both SNAP and BWA. STORMSeq took 10 h to complete the analysis of the exome using the BWA pipeline and 5 h using SNAP. When it took 176 h with BWA and 82 h with SNAP for genome analysis.

**Atlas2** is a downloadable package containing a suite of all the necessary tools for analyzing WES and WGS data produced from Roche 454, Illumina, and SOLiD platforms (Puri, Tiwary & Shukla, 2019). It is built using the Software-as-a-Service (SaaS) technology (Rumale & Chaudhari, 2017), which allows it to be deployed using two different cloud scenarios: local cloud and commercial cloud. Atlas2 relies on Genboree Workbench (Riehle et al., 2012) to be deployed in the local cloud. Genboree Workbench is a platform for deploying genomic tools as a service, that is currently being hosted at the Baylor College of Medicine. It offers a web-based interface, which allows scientists to interact with the pipeline, making it perfect for those with no bioinformatics background. Atlas2 relies on AWS to be deployed using a commercial cloud. It is built as an AMI image, and all it is required is to download it, upload the data to amazon S3 service and deploy it. Its operations comprise of a suite of variant detection software packages, which consists of Atlas-SNP2 for calling SNPs and Atlas-Indel2 for calling short indels. To demonstrate the effectiveness, authors tested it by performing two separate analysis: one with the Genboree Workbench platform and other with the AWS. In the Genboree test, authors sequenced the complete genomes of a two 14-year-old fraternal twins, both diagnosed with dihydroxyphenylalanine responsive dystonia (DRD), and proved that Atlas2 was able to successfully call all the relevant variants in both patients. In the AWS test, authors used one Illumina and one SOLiD file obtained from the 1,000 Genomes phase 1 project and demonstrated that it was able to successfully call all the variants. More importantly it only took 8–11 h to process the data, making it possible to perform WGS analysis on the cloud.

**Simplex** is a cloud-based pipeline for processing WES and WGS data (Fischer et al., 2012). It is built as a ready to use VirtualBox and a Cloud image, which allows users to deploy it quickly and easily in any compute infrastructure. Simplex has been developed for processing and analyzing short reads/sequences produced by the Illumina technology. Its operations include five steps: quality report, sequence alignment and refinement, alignment statistics, variant detection, and annotation and summary. In the quality report step, data is converted from Solexa and Illumina format into Sanger FASTQ. Then the data is checked for quality, and the results are reported. Afterwards, the data is then trimmed to a given read length and quality. Filters are later applied to eliminate errors, to reduce the number of variant false positives. To complete the quality report, step a second round of data quality statistic is generated, offering an overview of the quality improvements. In the sequence alignment and refinement step, two rounds of data alignment are performed to minimize the number of mismatching bases across all reads. BWA is used in the first round, and Genome Analysis Toolkit (GATK) is followed. The last operation in the sequence alignment step is to remove unmapped and improperly paired reads. The alignment statistics is the subsequent step, which evaluates the data quality before performing variant calling. Next is the variant calling step, in which INDELs and SNPs are called simultaneously. To carry out the variant calling processes the GATK tool is used. In the variant annotation and summary step, the variants are annotated using GATK and ANNOVAR (Wang, Li & Hakonarson, 2010). GATK is used to add annotation information from existing databases, such as RefSeq (Pruitt, Tatusova & Maglott, 2007), KEGG (Kanehisa et al., 2002), and dbSNP (Sherry et al., 2001) information. ANNOVAR is used to infer the functions of unknown variants. The results are then merged, and a summary report is outputted. Simplex has been evaluated by using data from the Kabuki study, (Ng et al., 2010) and demonstrates that they can analyze 42 samples simultaneously in a good timeframe. Furthermore, since the pipeline is free and opensource the pipeline is continuously tested and evaluated by multiple researchers around the world.

**Whole Exome sequencing Pipeline web tool (WEP)** is a centralized web-based application that can analyze WES data produced by Illumina platforms (D’Antonio et al., 2013). It offers a user-friendly web interface, designed for scientists without much knowledge of bioinformatics. It consists of three layers: submission, monitoring, and results. The submission layer validates the input files and deploys the pipeline. The monitoring layer provides information on the status of currently running pipelines and allows users to download the output data. The results layer graphs the outcome of the pipelines. The operations of WEP include six modules: Quality controls, Alignment, Conversion, Variant preprocessing, Variant calling and Postprocessing. In the quality control module, an overall quality check of the input data is performed, by using the FastQC tool (Leggett et al., 2013; Brown, Pirrung & McCue, 2017). The data is then passed to the NGS QC Toolkit (Patel & Jain, 2012) which filters low quality reads and removes primer/adaptor sequences. The alignment module is next, in which the data is mapped to a reference genome using BWA. The conversion module is followed, where data is converted and sorted by chromosomal coordinates by suing SAMtools (Li et al., 2009; Li, 2011). In the variant preprocessing module, the Picard tool (Liu et al., 2013) is used to mark and remove duplicate reads. The GATK tool is executed subsequently by performing local realignment, to improve the accuracy of variant calling and reduce false positives. The last step in this module is to call NGSrich which provides important information about the quality of data before variant calling is performed. In the variant calling module, WEP uses GATK tool, which allows to simultaneously call both SNPs and INDELs. The last module is the postprocessing, in which ANNOVAR, is used for annotating the variants. The output is then parsed and uploaded to a central database, and reports are generated. Since WEP is a free-to-use pipeline it has been validated by multiple researchers that have used WEP over the years.

**MC-GenomeKey** is a multi-cloud pipeline, which uses resources from different vendors (Google, AWS, Microsoft) to deploy a single pipeline, and minimize the overall cost of performing WES and WGS in the cloud (Ardagna, 2015). MC-GenomeKey consists of three features: Variant analysis, Workflow parallelization and cloud support. The variant analysis feature is comprised of four phases: quality check, read alignment, variant calling and variant annotation. In the first phase, data with low quality scores is trimmed out, this is achieved by using the FASTX-Toolkit (hannonlab.cshl.edu/fastx_toolkit/). In the second phase the data is mapped to the reference genome using the BWA tool. The variant calling phase is followed, in which the data is analyzed to determine where variants exist. It is accomplished by using the GATK tool. The variant annotation phase uses ANNOVAR to annotate all the variants found using knowledge from different structural and functional databases. Next feature is the workflow parallelization, which consists of a Python-based scheduling algorithm called Cosmos that constantly monitors the cost of the computing resources of multiple vendors. Based on that, it moves portions of the pipeline to cloud providers that offer cheapener resources, with the goal to minimize the overall cost processing NGS data in the cloud. The last feature of MC-GenomeKey is the cloud support, which allows scientists to deploy the MC-GenomeKey in two different cloud scenarios, which include individual cloud, and Multi-Cloud. Scientists have the option to deploy the entire pipeline in an individual cloud platform or deploy it across multiple cloud platforms. The authors tested MC-GenomeKey by using two datasets, a WGS and a WES dataset. The first experiment is to test the performance of MC-GenomeKey across different cloud provides, which included Amazon, Google and Microsoft. The results for both WGS and WES samples indicate that MC-GenomeKey can complete the pipeline faster when is deployed using Amazon resources and can be completed for cheaper when using Google resources. The second experiment involves the migration of portions of the pipeline to other cloud providers. Authors proved that using the migration model can lead to cost reduction in many cases with minor running time increases.

VDAP-GUI is a fully automated standalone pipeline, designed for non-IT scientists (Menon et al., 2016). It can analyze WES and WGS data produced by Illumina, Roche 454, and Ion torrent platforms. It is comprised of four steps: quality control and trimming, reference mapping and duplicate marking, variant calling, and annotation. In the first step, the quality and the read length of the data is analyzed using the FastQC tool, then it is filtered using the PRINSEQ (Schmieder & Edwards, 2011). In the reference mapping and duplicate marking step, the data is aligned to the reference genome using the BWA tool. Later the data is subject to duplicate marking using the Picard tools to remove false positives. The variant calling step is followed, which performs a technique called MultiCom where three variant calling tools are simultaneously executed and only variants that appear in at least two of them are selected. VDAP-GUI relies on SAMtools, VarScan (Koboldt et al., 2009) and Freebayes (Garrison & Marth, 2012) to perform the variant calling. In the final step variants are filtered and annotated using the VEP tool. Authors validated VDAP-GUI using a publicly available human WES dataset PDA_033-Tumor. It was able to detect a total of 55,919 SNPs, and in addition, it was able to detect 46,963 SNPs that were not reported in the previous study, solidifying the validity and the effectiveness of VDAP-GUI.

**Fastq2vcf** is a standalone pipeline for processing WES and WGS data (Gao, Xu & Starmer, 2015). It integrates multiple sequencing analysis tools to achieve better data processing efficiency. Fastq2vcf is built around three shell script files: “QC_mapping.sh”, “PreCalling.sh” and “Variant.sh”. The QC_mapping script performs quality control of the raw data, by using the FastQC tool, which provides a summary of the quality of the data processed. The next is to align the data with the reference genome, by using the BWA tool. The PreCalling script subsequently removes duplicates from the data, to improve the quality of the variant calling, by using the MarkDuplicate command-line tool from Picard. Then GATK recalibration tools are used to perform local realignments and base quality recalibration to help correct the misalignments. Finally, the last script performs variant calling, which uses four tools simultaneously: GATK UnifiedGenotyper, GATK HaplotypeCaller, SAMtools and SNVer (Wei et al., 2011), and the outputs of all the tools are consolidated into a single call set. Only the variants that appear in all of them are used. Lastly ANNOVAR, and VEP are used to annotate the variants. Authors evaluated Fastq2vcf by using a five WES sample, and demonstrated the effectiveness of Fastq2vcf by deploying it on commodity hardware and discovered a total of 55,919 SNPs while only taking 27 h to compute the results.

**ExScalibur** is a set of high-performance cloud-based pipelines for processing WES data (Bao et al., 2015). Authors have designed ExScalibur for processing and analyzing short reads/sequences produced by the Illumina technology. Its operations include seven modules: quality control, preprocessing, alignment, alignment refinement, variant calling and filtering, annotation, and project report generation. In the first module the raw sequencing reads are assessed for base quality, duplication level, and nucleotide composition. The preprocessing module is followed, where adapters and removed. Next, is the alignment module, which maps the raw sequence reads into the reference genome using three different aligners: BWA-aln, BWA-mem, and Novoalign. Later, the alignment refinement module improves the alignment by using the base quality score recalibration tool. Subsequently the variant calling module, performs parallel execution of multiple variant callers to increase the variant calling confidence. The variant callers are split into two groups germline and somatic. The germline callers include GATK UnifiedGenotyper, GATK HaplotypeCaller, FreeBayes, SAMtools mpileup/bcftools, Isaac Variant Caller (Raczy et al., 2013) and Platypus (Rimmer et al., 2014). The Somatic variant callers include MuTect (Cibulskis et al., 2013), Shimmer (Hansen et al., 2013), SomaticSniper (Larson et al., 2012), Strelka (Saunders et al., 2012), VarScan2 (Koboldt et al., 2012) and Virmid (Kim et al., 2013). The variant filtering module is followed where some custom build filters are applied, to remove false positives. Lastly variants are annotated for gene symbol, functional changes, population frequency, and a comprehensive data analysis report is generated, offering statistics on the variant calling processes. Authors validated ExScalibur by using two different types of WES datasets: germline mutations (GMD) and somatic mutations (SMD) datasets. Each of the datasets where generated using two techniques, simulation, and real data. The simulated dataset was created using the Genome in a Bottle Consortium from the genome of NA12878. In the real dataset they used 30 Acute Myeloid Leukemia (AML) tumor/normal pairs (Cibulskis et al., 2013). Each dataset was tested using a combination of aligners and callers, including a one where two aligners and two callers were performed at the same time. For the GMD experiments on the simulated data authors reported achieving a sensitivity of 99.03% when using two aligners and two callers. Similarly, for the AML dataset, authors achieved a 98.03% sensitivity when using the two aligners and callers. In the SMD experiments on simulated data authors achieved 90% sensitivity for all different combinations of aligner and callers. Similarly, results were obtained for the AML dataset, suggesting that using of several aligner and caller variants can be detected with a higher confidence.

**DNAp** is docker container that users download onto their systems and contains all required tools for analyzing WES and WGS data (Causey et al., 2018). It requires necessary computer hardware to execute it, including a minimum of 1.5TB for locally saved inputs and outputs in the working directory. It consists of five steps: quality control, alignment, re-alignment, bam quality control, variant calling, and annotation. The first step validates the quality of the FASTQ files by using the FastQC tool, next is alignment, in which the data is aligned to the reference genome using the BWA tool. The data is then sorted, index, and duplicates are marked and removed using the Picard tools. The output is then merged into a single file by using the MergeSamFiles tool from Picard. The realignment step is followed, in which the data is re-aligned to reduce the number of variant false positives, to do that the GATK toolset is used. The subsequent steps of the pipeline vary depending on the type of the data that is being processed. When working with WES data the bam quality control step uses GATK diagnoseTargets and the Qualimap tool to examine the sequencing alignment data. When processing WGS data in the bam quality control step uses GATK DepthofCoverage tool. Variant calling is followed, and just like the previous step, when consuming WES data, it uses MuTect2 and Strelka. When processing WGS data it uses BreakDancer and Lumpy. Laster, a consensus algorithm is executed to find the variances that are common in both callers, this is done to reduce the number of variant false positives. In the final step the variants are annotated using the Snpeff (Cingolani et al., 2012) and Oncotator (Ramos et al., 2015) tools. DNAp was evaluated using two different datasets: a human and a mouse. In the human dataset, it was able to identically call 90% of the variances for the human dataset, when compared to the reference results. In the mouse dataset, it was able to identically call 89.8% of the variances present in the reference dataset. Furthermore, an additional test was performed to show that the pipeline could be deployed in different heterogeneous platforms and the same results were obtained.

**SeqBench** is a centralized web-based application that merges the management and analysis of WES and WGS data into a single application and combines it with an easy-to-use interface to facilitate the data handling (Dander et al., 2014). It is organized into three different modules: data acquisition, the dashboard, and the visualization. The data acquisition module validates the input data provided and deploys the pipeline. The dashboard lays out the status and the statistics of deployed pipelines. Lastly, the visualization module, displays the results obtained. Authors of SeqBench highlight the fact that SeqBench is built on top of an already evaluated and validated cloud-based analysis pipeline named Simplex. For those reasons, no further results are reported.

### Pros and cons of reported WGS/WES pipelines

Meeting our earlier discussed survey and review methodology, in this manuscript, we have investigated and reported important elements of eleven different state-of-art WGS and WES pipelines, published with in last ten years and made available through PMC/ NCBI. We have compared and highlighted common and variable features of these pipelines in Table 1, and those include, interactive and user friendly graphical interface; open-source code and freely available to the community; can be deployed as a standalone application; able to process multi samples at a time (parallel processing); support debugging; able to process both or individual WES and WGS data; check data quality; generate data quality reports; perform alignment, remove duplicates, call and annotate sequence variant; support long reads-based data processing; provide data simulation and visualization; automatic variant data extraction, transfer and loading into database management system; support SQL based data manipulation; and integration with annotation databases.

**Table 1:** Feature comparison of STORMseq, Atlas2, Simplex, WEP, MC-GenomeKey, VDAP-GUI, fastq2vcf, ExScaliburn, DNAp, and SeqBench pipelines. The table compares following pipeline features: Interactive and user-friendly graphical interface; Open-source code and freely available to the community; Deployment as a standalone application; Able to process multi samples at a time (parallel processing); Support debugging; Able to process WES data; Able to process WGS data; Generate data quality reports; Check Data Quality; Align to reference genome; Remove Duplicates; Call Variants; Annotate Variants; support long reads-based data processing; provide data simulation and visualization; automatic variant data extraction, transfer and loading into database management system; support SQL based data manipulation; integration with annotation databases.

Features/ Pipelines	STORMseq	Atlas2	Simplex	WEP	MC-GenomeKey	VDAP-GUI	fastq2vcf	ExScaliburn	DNAp	SeqBench
Interactive and user-friendly graphical interface	Yes	Yes	Yes	No	Yes	No	No	Yes	Yes	No
Open-source code and freely available to the community	Yes	Yes	Yes	Yes	Yes	Yes	Yes	Yes	Yes	Yes
Deployment as a standalone application	Cloud	Cloud	Cloud	Centralized	Cloud	Standalone	Standalone	Cloud	Cloud	Centralized
Able to process multi samples at a time (parallel processing)	Yes	Yes	No	No	Yes	Yes	Yes	Yes	Yes	Yes
Support debugging	No	No	No	No	Yes	No	No	Yes	No	No
Able to process WES data	Yes	Yes	Yes	Yes	Yes	Yes	Yes	Yes	Yes	Yes
Able to process WGS data	No	No	Yes	No	Yes	Yes	Yes	No	Yes	No
Generate data quality reports	No	Yes	Yes	Yes	Yes	Yes	Yes	Yes	Yes	Yes
Check Data Quality	Not reported	Not reported	Fastx	FastQC, Ngs-qc	Fastx	FastQC	FastQC	Not reported	FastQC	Fastx
Align to reference genome	BWA	Not reported	BWA	BWA	BWA	BWA	BWA	BWA	BWA	BWA
Remove Duplicates	Not reported	Not reported	Not reported	Picard	Picard	Picard	Picard	Picard	Picard	Not reported
Call Variants	GATK	Atlas-SNP2, and Atlas-Indel2	GATK	GATK	GATK	SAMTools,VarScan,FreeBayes	GATK, SAMtools, and SNVer	GATK, FreeBayes, SAMtools,Isaac Variant Caller, and Platypus	MuTect2, Strelka, BreakDancer, Lumpy	GATK
Annotate Variants	VEP	Not reported	GATK, ANNOVAR	ANNOVAR	ANNOVAR	VEP	ANNOVAR and, VEP	ANNOVAR	SnpEff, and Oncotator	GATK, ANNOVAR
Support long reads-based data processing	Not reported	Not reported	Not reported	Not reported	Not reported	Not reported	Not reported	Not reported	Not reported	Not reported
Provide data simulation and visualization	Not reported	Not reported	Not reported	Not reported	Not reported	Not reported	Not reported	Not reported	Not reported	Not reported
Automatic variant data extraction, transfer and loading into database management system.	Not reported	Not reported	Not reported	Not reported	Not reported	Not reported	Not reported	Not reported	Not reported	Not reported
Support SQL based data manipulation	Not reported	Not reported	Not reported	Not reported	Not reported	Not reported	Not reported	Not reported	Not reported	Not reported
Integration with annotation databases	Not reported	Not reported	Not reported	Not reported	Not reported	Not reported	Not reported	Not reported	Not reported	Not reported

In our conclusions, not all developed and reported pipelines are interactive, based on user friendly graphical interface and can be used by the users without strong computational and programming background. According to our review, STORMseq, Atlas2, Simplex, MC-GenomeKey, ExScaliburn and DNAp are claimed by their authors to be user friendly pipelines but still these cannot be easily deployed and applied for the processing of WES/WGS data. However, all discussed WGS/WES pipelines in this manuscript are open-source code and freely available to the community, and able to process single (sequential) and multi samples (parallel processing) at a time. VDAP-GUI and fastq2vcf are standalone, WEP and SeqBench are recommended for centralized computational environment, when remaining all pipelines (STORMseq, Atlas2, Simplex, MC-GenomeKey, ExScaliburn and DNAp) are cloud based (*e.g*., AWS supported and tested). With these, current and major limitations of cloud-based pipelines include affordability (high cost) and unsupervised deployment for data processing. Standalone and centralized environment-based pipelines require creating reference indexes and integration of compatible tools used in the published pipeline. Any change in those do not support successful execution of pipelines. One another important aspect is debugging, and except MC-GenomeKey and ExScaliburn, none of the discussed pipeline supports explicit debugging. All mentioned pipelines can process WES data, however, authors of STORMseq, Atlas2, WEP, ExScaliburn, SeqBench have not mentioned using these for WGS data.

Except STORMseq, all reviewed pipelines explicitly support quality checking of WGS/WES data, and most wide used software application proved to be the FastQC. Other than Atlas2, all pipelines are using BWA for mapping sequences to the reference genome. Except STORMseq, Atlas2, Simplex and SeqBench, all have applied Picard tools for removing duplicates. GATK is used by the for calling variants, the STORMseq, Simplex, WEP, MC-GenomeKey, fastq2vcf, ExScaliburn, and SeqBench. When Atlas2 applied Atlas-SNP2 and Atlas-Indel2; VDAP-GUI used SAMTools, VarScan, FreeBayes; and DNAp implmented MuTect2, Strelka, BreakDancer and Lumpy. To annotate variants, STORMseq, fastq2vcf, and VDAP-GUI used VEP; Simplex and SeqBench applied GATK; and WEP, MC-GenomeKey, fastq2vcf, ExScaliburn, and SeqBench have implemented ANNOVAR. When, Atlas2 have not declared and only DNAp has used SnpEff, and Oncotator.

During our review and comparative analysis, we found none of these pipelines have been recommended to process long reads based WGS/WES data (*e.g*., generated using PacBio and Oxford nanopore) but only for short reads, mainly generated using Illumina sequencing technology. Data simulation and visualization is a key to perform and report efficient downstream analysis, when all these pipelines do provide any kind data visualization supporting variant data interpretation and annotation. Today, when big data is well supported with the used database management systems and implementation of SQL for data manipulation, all discussed pipelines do not provide any kind of automated variant data extraction, transfer and loading process. Furthermore, outcome of these pipelines cannot be straight forwardly integrated with any of the available annotation databases for timely interpretation and critical analysis. These all limitations do not support application of these pipelines in clinical settings and users from variable backgrounds cannot easily apply these for timely WGS and WES data processing, analysis, simulation, interpretation, and reporting of reproducible results in unorthodox computation and clinical settings.

Precision medicine requires development of progressive healthcare environments that incorporate heterogeneous genomic data into clinical settings. None of these genomics pipelines can efficiently incorporate and leverage genomic (WGS/WES) data processing and analysis in clinical settings, and support decision-making. Furthermore, processed variant data through these pipelines is not available in Artificial Intelligence and Machine Learning (AI/ML) ready formats. So, it can be directly used for integrated and predictive analysis, and deep phenotyping.

## Discussion

The first DNA sequencer came out in 1977 by the Sanger (Sanger, Nicklen & Coulson, 1977), and later new NGS technologies and methods emerged with time. The chemical structure of the genome is double-stranded DNA, and the smallest unit of genetic information is the base pair (bp), which is two nucleotides paired by hydrogen bonds across the double helix (Langridge et al., 1957; Chargaff, 1979; Laird, 1971). DNA stands (polynucleotides) are composed of four smaller chemical molecules called nucleotides: adenine (A), cytosine (C), guanine (G), and thymine (T) (Chaffey, 2003). The total information content in a haploid set of chromosomal DNA is approximately 3.2 billion bp (Chial, 2008). The human genome consists of about 3 × 109 base pairs of DNA. The distance between bases along the DNA strand is ≈ 0.3 nm (1 billionth of a m) (Marvin et al., 1961), the length of the fully stretched out human DNA molecule is ≈3 Giga-bp (billion bp), therefore, each cell harbors roughly 1 m of DNA, and remarkably, each cell in our body must compress this one-meter-long DNA into a nuclear volume with a radius of only a few microns. The majority (≈62%) of the human genome comprises of intergenic regions (Nelson, Hersh & Carroll, 2004; Bartonicek et al., 2017), the non-protein coding (Clamp et al., 2007) parts of the genome that lie between genes (Djebali et al., 2012). These intergenic sequences used to be called “junk DNA”, (Pennisi, 2012; Wright & Bruford, 2011; Palazzo & Lee, 2015) but now genome research over the last few years has revealed functions associated with these regions, suggesting that every part of the genome may have some importance. The bulk of the intergenic DNA is composed of transposable elements, randomly distributed repeated sequences called interspersed repeats (Smit, 1999; Smit, 1996), and closely spaced repeat units called tandemly repeated repeats (Quilez et al., 2016; Press, Carlson & Queitsch, 2014) DNA. Intergenic DNA may also include gene regulatory sequences (Levo & Segal, 2014; Sheffield & Furey, 2012) such as promoters (Levine, 2010; Williamson, Hill & Bickmore, 2011), enhancers (Tirosh & Barkai, 2008; Aow et al., 2013), and silencers (Bushey, Dorman & Corces, 2008) that have yet to be characterized. The functional elements of the genome are discrete DNA segments that encode a defined product or display a reproducible biochemical signature (*e.g*., protein-binding or a specific chromatin structure). Segments of DNA that carry genetic information are called genes (Maglott et al., 2005; Brown et al., 2015; Gerstein et al., 2007; Durmaz et al., 2015; Portin, 2002; Cavalli-Sforza, Menozzi & Piazza, 1996). A gene is a hereditary unit of DNA sequence transferred from parent to offspring that defines a biological function. Alteration to gene is known as mutation/variant, and WGS/WES are most adopted sequencing methodologies to investigate it.

Even with immense molecular and computational advancements, still the goals of completely discovering and understanding human genome functions have not been fulfilled (Chen et al., 2019). There are quite a few publicly and commercially available tools that support interpretation of sequence variants (Zeeshan et al., 2020; Ahmed et al., 2019; Ahmed et al., 2020b). Many predictive algorithms (Ahmed et al., 2020a) have been proposed and implemented to determine effect of the variant on the primary and alternative transcripts, and impact on protein (Richards et al., 2015). These algorithms are mainly divided among three types: Missense (Thusberg, Olatubosun & Vihinen, 2011), Splice site (Jian, Boerwinkle & Liu, 2014), and Nucleotide conservation (Savas et al., 2004). Missense prediction includes but not limited to: ConSurf (Ashkenazy et al., 2016), Functional Analysis Through Hidden Markov Models (FATHMM) (Shihab et al., 2013), MutationAssessor (Gnad et al., 2013), PANTHER (Thomas et al., 2003), PhD-SNP (Capriotti & Fariselli, 2017), Sorting Intolerant From Tolerant (SIFT) (Ng & Henikoff, 2003), SNPs&GO (Capriotti et al., 2013), Align GVGD (Hicks et al., 2011), MAPP (Chao et al., 2008), MutationTaster (Hombach et al., 2019), MutPred (Pienaar, Howell & Elson, 2017), PolyPhen-2 (Adzhubei et al., 2010), PROVEAN (Choi & Chan, 2015), nsSNPAnalyzer (Bao, Zhou & Cui, 2005), Condel (González-Pérez & López-Bigas, 2011), and CADD (Rentzsch et al., 2019). Splice site prediction includes, GeneSplicer (Leman et al., 2018), Human Splicing (Spurdle et al., 2008), Finder (Tang, Prosser & Love, 2016), MaxEntScan, NetGene2, NNSplice, FSPLICE. When, Nucleotide conservation predication are made using Genomic Evolutionary Rate Profiling (GERP) (Shamsani et al., 2019), PhastCons (Anna & Monika, 2018), PhyloP (Moles-Fernández et al., 2018). To support variant interpretation and annotation, there are some commercial and freely available reference databases (*e.g*., Ensembl, GenCode, ClinVar, GeneCards, DISEASES, HGMD, OMIM, GTR, CNVD, GWAS Catalog, COSMIC, dbGaP etc.). These have been designed to support the storage and sharing of sequence data of different types (*e.g*., genes, somatic and germline mutations etc.), species (*e.g*., human, mouse, canine, fish, etc.) and size (Koboldt et al., 2010; Cavalleri & Delanty, 2012; Pepin et al., 2016).

Processed high quality WGS/WES data (*e.g*., generated by Illumina HiSeq) concludes with, if not millions then over hundred thousand variants (Abecasis et al., 2010). Downstream analysis of dataset including few samples can be well managed by the small team of bioinformaticians. However, investigating susceptibility of multiple samples (*e.g*., hundred/thousands) is cumbersome, tedious and time consuming. It is still a challenging task today to perform automatic downstream analysis, which includes gene-variant discovery, annotation, prediction, and genotyping. Furthermore, it is difficult to timely detect *D. Novo* Single-Nucleotide Variants (DNSNVs) (Liang et al., 2019) and minimize the number of false negatives (Hwang et al., 2019). Implementing platforms dealing big data analytic challenges require manpower (*e.g*., bioinformaticians, biostatisticians), computational resources (*e.g*., HPC and cloud computing environments), and bioinformatics applications (*e.g*., data inspection, mapping to reference genomes, expression analysis and variant calling). In this manuscript, we have reported our analysis of different genomics applications designed and developed to process and analyze WGS/WES data. We have discussed all applications individually, as well as, performed features based comparative analysis, which includes quality checking (QC), alignment to reference genome, removing duplicates, variant calling, and gene-variant annotation. Most popular bioinformatics applications include FastQC and Fastx are for quality checking, BWA for alignment a reference genome, Picard for removing duplicates, GATK for variant calling, and ANNORVAR and VEP for annotation. We have also assessed the user friendliness, open source and freely availability, deployment environment types, multi sample processing at one time, and debugging and troubleshooting support. We were interested to find if applications can be used by the scientists without much computer science knowledge and bioinformatics skills; allow processing of multiple WGS/WES samples at the same time; report results and log each computational step involved in pipeline.

We need to implement personalized approaches to improve the traditional symptom-driven medical practice with disease-causal genetic variant discovery Ahmed et al. (2021). Major challenges need to be addressed include but not limited to: developing disease-specific cohorts based on patients’ genomics profiles; searching for the underlying immunity genes, and common and the rare disease-causal variants, imputations, and haplotype resolutions; finding variant’s agnostic of certain predetermined pathologies – like, similarities between disease states not classically considered related at a molecular level; and predicting genetic variants not only affecting targeted but other disorders in particular subjects, which can then be extended to the overall population. Furthermore, it is also important to address ethical issues related to genomics data and those include, forensic identification, and personal, family, and ethnic identities; rights of lost privacy; genetic discrimination; misused human genomics; state-enforced eugenics; and ownership and control of genetic information of participants (Takashima et al., 2018; Roche, 2009; Niemiec & Howard, 2016; Ahmed & Shabani, 2019). We need to implement Findable, Accessible, Intelligent, and Reproducible (FAIR) methodologies to efficiently process and analyze high volume - heterogeneous genomics data with multiple data structures in real-time.

## Conclusions

In this manuscript, we have evaluated and reported different genomics pipelines developed to investigate susceptibility of WGS/WES data for variant discovery, annotation, prediction, and genotyping. During our evaluation of discussed pipelines, we found that most of the pipelines are not user friendly and require programming and scripting skills. Furthermore, none of these supports efficient high-volume variant data management and visualization to support timely downstream analysis with less manual and computational power.

## List of Abbreviations

AI: Artificial Intelligence
AML: Acute Myeloid Leukemia
AMI: Amazon Machine Image
AWS: Amazon Web Services
BWA: Burrows-Wheeler Alignment
DB: Database
DBMS: Database Management System
DNSNVs: *De novo* single-nucleotide variants
DNA: Deoxyribonucleic acid
DRD: Dihydroxyphenylalanine Responsive Dystonia
ERD: Entity Relationship Diagram
ETL: Extract Transform Load
FAIR: Findable, Accessible, Intelligent, and Reproducible
FATHMM: Functional Analysis Through Hidden Markov Models
GEO: Gene Expression Omnibus
GATK: Genome Analysis Toolkit
HPC: High-Performance Computing
INDEL: Insertion or deletion
IGV: Integrative Genomics Viewer
ML: Machine Learning
NGS: Next Generation Sequencing
ONT: Oxford Nanopore Technologies
PE: Paired End
QC: Quality Check
SOLiD: Sequencing by Oligonucleotide Ligation and Detection
SAM: Sequence Alignment Map
SRA: Sequence Read Architecture
SIFT: Sorting Intolerant From Tolerant
SE: Single End
SNP: Single Nucleotide Polymorphism
SMS: Single Molecule Sequencing
SMRT: Single Molecule Real Time
SaaS: Software-as-a-Service
VCF: Variant Call Format file
WES: Whole Exome Sequencing
WEP: Whole Exome Sequencing Pipeline
WGS: Whole Genome Sequencing

## Additional Information and Declarations

### Data Availability

The following information was supplied regarding data availability: This is a review article. Data and source code are not applicable. However, the source code of discussed applications (where applicable) is already made available online by the developers.
